# Hip Diseases

**Published:** 1855-05

**Authors:** 


Hip Disease.—The affections which bear more or less resemblance to hip disease are, psoas abscess, caries of the pelvis, inflammation of the sacro-ilias articulations, chronic rheumatism of the hip-joint, sciatica, and hysterical affections of the joint.

You are already aware how, in the early stage, hip disease seldom presents any other symptoms than pain and some slight impediment to motion, and hence you can see at once that nervous disorders are the most likely to simulate this first stage of the disease, because their characteristic sign is pain, and the pain, if severe, entails more or less difficulty of motion. What, then, are these painful nervous disorders? They are sciatica and hysterical affections of the joint. Sciatica is chiefly characterized by pain, so is hip disease, at the commencement, but there are several circumstances by which the two complaints can be distinguished without much difficulty. The pain in sciatica becomes very severe soon after the onset of the disease, and though it may be first felt about the hip it soon extends down the thigh to the leg, following the course of the sciatica nerve and its branches. In hip disease, as I have told you, the pain as often commences in the knee as in the hip, perhaps oftener; it is seldom very acute at first, and artificial motion during this early stage never produces the violent aggravation of pain which is excited by moving the limb during a severe attack of sciatica. Again, patients affected with hip disease can go about tolerably well during the first stage, showing slight lameness only, whereas a person attacked by sciatica is soon laid up and quite unable to place the foot on the ground, much less bear the weight of the body. As the diseases progress their distinguishing symptoms become much more clear and decided. After the first smart attack, sciatica has a tendency to diminish, even though no treatment be employed; mere rest alleviates the pain, and in a week or two a manifest remission takes place, whereas the progress of hip disease is steady and onwards; and during the second or third stages no error of diagnosis can be committed.

Hysterical affections of the joint simulate incipient hip disease much more closely. Like sciatica, these affections are characterized by intense pain in the neighborhood of the joint, together with a real or imaginary inability to move the affected limb; but on carefully examining all the circumstances of the case, you will seldom experience any difficulty in arriving at an accurate diagnosis. In the first place, the patients are almost invariably females; and whenever one of the fair sex complains of intolerable pain in a joint, with little appearance of organic lesions beyond a mere puffiness of the integuments, you may be pretty certain that her complaint is hysterical, for it has been calculated that at least four-fifths of the females amongst the higher classes of society, supposed to be suffering from diseases of the joints, are, in reality, affected with hysteria, and nothing else. You will not, however, rest content with this general inference, but direct your attention to the concomitant signs of hysteria, and compare any existing symptoms with those that are known to attend true hip disease at a corresponding stage. It is an established fact, that hysterical females are more subject than any other individuals to neuralgia, and it seems to me highly probable that the complaint now under consideration is hysterical neuralgia of the joint. Many of the characters of the pain lead to this conclusion. It is excessively violent from the commencement, and in this respect differs from the pain of true morbus coxarius. The patient also refers it to the hip-joint, and not to the knee, as is so often the case when the disease depends on incipient organic changes. The pain, again, is not limited to the affected articulation, but extends to the buttock and down the thigh, a circumstance in which it resembles sciatica. This last affection, indeed, is often nothing but rheumatic neuralgia. This general diffusion of the pain constitutes an useful distinction between the hysterical complaint and true disease of the joint. The irregularity of the pain, and the peculiar modifications of sensibility which accompany it, are highly characteristic, and at once point to an hysterical origin. The pain, though apparently intense, is not constant; it often suddenly disappears for a time, without any evident cause, particularly if the patient's mind be diverted, from her condition; and in like manner, the more the sufferer's attention is directed to the part, the more she complains and appears to suffer. Besides this, the pain is absent during sleep, whereas the rest is always more or less disturbed by the pain of organic disease. The sensitiveness of the skin is truly hysterical; the patient shrinks from the approach of the finger, yet sometimes will allow you to press the joint in all directions, without complaining of much pain. The nates and the limb in general are not wasted, and there is no elongation of the limb. Sometimes, however, from the spasmodic muscular contractions to which these hysterical patients are very liable, the limb is drawn up and inverted, the trochanter major is prominent, the knee bent, and the thigh rests on the sound one. Here the case might, by a superficial observer, be easily mistaken for dislocation; but its history, and the possibility of bringing the affected limb into a natural position, will distinguish the case from one of true displacement of the bone.

The foregoing are examples in which functional disorders simulate organic disease in its early stages; in other cases, the difficulty of diagnosis may depend on the existence of some organic lesion in or near the joint, yet differing in its nature from the changes which constitute true hip disease.

It is evident that the more nearly these lesions resemble in their nature those of morbus coxarius, and the closer they are to the joint, the more numerous will be the points of resemblance between the two affections. Experience proves this to be the case, and shows that caries of the pelvis, inflammation of the sacro-iliac symphysis, and psoas abscess, are the affections most likely to be confounded with chronic hip disease. When caries affects any portion of the pelvis close to the hip-joint, the functions of the articulation are impeded; pain is felt in the region of the joint, and abscesses form in its neighborhood; but the general symptoms are always less severe than when the articular cavity is the seat of the disease; the pain, also, is less severe, and not so evidently located in the joint, the movements of which are not very materially impeded. In hip disease, the immobility of the limb, after a certain period, is a constant and characteristic symptom; whereas here, although the hip-joint may be stiff, and a disinclination to movement exist, certain motions can be affected, without inducing any great pain. The suppuration from caries of the pelvis is less abundant in most cases than the discharge from hip disease, and after some time the probe will detect the existence of caries in the bone, when all doubt as to the nature of the disease will be removed.

Chronic inflammation of the sacro-iliac symphysis often bears a close resemblance to morbus coxarius. In children, a true joint, furnished with its synovial membrane, exists at the union between the sacral and iliac bones, and the inflammation is of the same scrofulous character as that which attacks other bones at an early period of life. The symptoms follow the same slow and progressive course as in hip disease. At first the patient complains of pain in the lumbar region; in some cases the pain is seated quite close to the hip, and is referred to that joint; or it may show itself from the commencement in the knee—a circumstance very likely to deceive the practitioner, and induce him to refer the symptom to disease of the hip-joint. Lameness soon follows, and the symptoms, which, as you perceive, are the same as those observed at the commencement of hip disease, gradually increase in intensity. But this is not all; they soon give rise to other symptoms also, analogous to the effects produced by disease of the hip. Thus it will be observed the patient avoids lying on the affected side; the nates are flattened and altered in shape; the muscles of the limb become wasted; the patient throws his weight on the sound limb; the affected one is apparently elongated, from depression of the pelvis on the affected side; the knee is slightly flexed, and the point of the foot directed either forwards or slightly outwards. As the disease progresses, the symptoms increase, and the elongation of the limb is followed by shortening, which adds another remarkable point of resemblance between this disease and true coxalgia. If not arrested at this stage, abscesses form in the neighborhood of the posterior spinous process of the ilium, and open either on the back of the thigh or in the groin; the suppuration becomes abundant, and the patient is worn out with hectic. This unfavorable termination is, however, more rare than in hip disease, for the progress of the malady is arrested, or imperfect anchylosis takes place between the opposite surfaces of the iliac and sacral bones.

From this account, you will perceive many points of resemblance between the two affections: we have in both pain and lameness at the commencement; we have the same, or nearly the same, appearances of the nates and limb, the same position, the same lengthening followed by shortening, and the same formation of fistulous abscesses about the upper part of the thigh. Great attention will therefore be required to distinguish inflammation of the sacro-iliac symphysis in a young subject from hip disease, not only at its commencement, but in subsequent stages. The first point to which attention should be directed is the precise seat of the pain. In hip disease, the pain of the joint, when it exists, is most severe in the groin, or just behind the trochanter major; in the other affection, the pain is chiefly developed by making pressure transversely near the posterior superior spinous process of the ilium. When tumefaction exists, its seat is also different. In hip disease, the fullness is at first perceptible either in front of the joint or immediately behind the trochanter major; in chronic inflammation of the sacro-iliac symphysis, we never discover any tumefaction in the groin, but the puffiness or swelling is always seated over the posterior spine of the ilium. In hip disease, the joint soon becomes fixed, and any attempted motion excites intense pain, whereas the thigh can be freely moved while the patient is lying down, in cases of sacro-iliac affections. The limb, in cases of this latter disease, can be extended without much difficulty; and although the patient may walk very lame, he puts the whole of the foot on the ground; while, in hip disease, the toes only touch the ground, and the heel is elevated. Finally, the shortening of the limb in sacro-iliac affections is always apparent, not real; but in hip disease, the shortening depends on destruction of the acetabulum and head of the femur, or on dislocation of the dorsum ilii; it is real, instead of being apparent.

Formation of matter about the region of the groin or hip, accompanied by pain and difficulty of motion, might lead to the inference of disease of the hip joint in its third stage, and cases are recorded where psoas abscess has been mistaken for it; but attention to the following points will always enable you to distinguish the one complaint from the other. In psoas abscess the pain commences in the lumbar region, and is not felt in the groin until the disease has made considerable progress. Rotation outwards of the limb greatly increases the pain whereas the reverse is noticed in cases of diseased hip-joint; the various positions of the limb, its elongation, shortening, &c., which characterize morbus coxarius, are not observed in psoas abscess, and the peculiar effect of position, of coughing, &c., in increasing the swelling or discharge of matter depending on psoas abscess, is altogether absent in cases of hip disease.

There are still to be noticed two series of cases, one of which may simulate generally disease of the hip, while the other only resembles the third stage of that affection. The complaints to which I now allude are a peculiar shortening of the limb, produced by injury to the hip-joint, and chronic rheumatism, or, as it has been otherwise called, morbus coxæ senilis. It was formerly supposed that all cases of real shortening of the limb depended either on the hip disease which I have thus described, on fracture within the capsule, or on chronic rheumatism. But Mr. Gulliver has described, in the *Edinburg Medical and Surgical Journal*, (No. 128, vol. xlvi. p. 97,) some cases of shortening of the neck of the thigh-bone, depending upon other causes, and which are particularly worthy of attention. This peculiar lesion has been hitherto attributed either to fracture within the joint, or to the natural effects of old age; but Mr. Gulliver has clearly shown that it may occur in young subjects, as the result of injury to the joint, and under circumstances which give considerable resemblance to the latter stage of hip disease. The history of these cases, as related by Mr. Gulliver, may be comprised in a few words. A young man receives an injury to the hip, from a fall probably, and immediately afterwards complains of pain in the joint. The pain is not very severe, but it continues, and lameness supervenes; these symptoms may go on for many months, the thigh wasting, and the limb slowly shortening, until it becomes perhaps two or three inches shorter than the sound limb.

Cases of the kind described by Mr. Gulliver bear a close resemblance to hip disease in its earlier stage, and at the onset cannot well be distinguished from it; but as the malady progresses the differential signs soon become evident, and a little attention will enable us to discover them. Although the limb be a couple of inches shorter than the sound one, and although the shortening be found to depend on organic lesion, the motions of the joint are perfectly free. Now this could never occur in ordinary hip disease, and the reason why it happens in the cases now alluded to is that the synovial membrane and cartilages are quite free from disease. For the same reason also there is little or no pain produced when the opposite surfaces of the joint are brought into contact by pressing the head of the femur against the acetabulum; there is no tumefaction about the joint, abscesses never form, and the patient remains free from constitutional disturbance of any kind, although the limb is continually getting shorter and the lameness increasing until it becomes incurable. All these circumstances are incompatible with the notion of chronic hip disease, such as I have described it to you; and even at the earliest stage, while the pain and lameness are considerable, we cannot detect on examination any signs of inflammation going on within the joint. I should also mention that the patient, though he limps, puts the foot flat on the ground—another point of distinction between this disease and morbus coxarius.

A short account of the pathology of these interesting cases will enable you to understand better the symptoms by which they are accompanied. The synovial capsule of the joint, and the articular cartilages, are quite healthy; but the neck of the thigh bone is much shorter than is natural, and forms nearly a right angle with the shaft; or the neck may be removed altogether by absorption, while the head is flattened and expanded. On dividing the parts, the shell of the neck if any remains is found condensed, and, in one case, the cancelli contained caseous matter. Sometimes the acetabulum is widened and remarkably shallow, corresponding to the alteration of shape in the head of the bone. These cases have been assimilated to the examples of interstitial absorption of the neck of the femur which takes place as a natural consequence of old age; but, in my opinion, they have a different origin, and should be referred to absorption—the result of osteitis without any tendency to suppuration.

Analogous, in outward appearance at least, to the foregoing affection, is the chronic rheumatism of the hip-joint, which has been well described by Mr. Robert Smith and Mr. Adams of Dublin. This disease generally affects patients above fifty years of age, though it may occur at an earlier period of life. Its pathological characters are peculiar. The cartilage of the head of the femur is more or less destroyed, not by ulceration, but by a process which seems analogous to interstitial absorption; it looks, in fact, as if it had been worn away by the effects of long continued friction; and the points at which the cartilage is most extensively removed correspond to this notion of wearing down or *usure*, as the French surgeons term it. The neck of the femur is also shortened to a greater or lesser degree, and sometimes so much so that the change produced bears a strong resemblance to fracture of the neck of the bone within the capsule. The os femoris is expanded and flattened, while the acetabulum, which is likewise deprived of its cartilage, is rendered wider and deeper than natural. When the disease has continued for any length of time, the round ligament disappears, and the osseous surfaces, from which the cartilages have been removed, are covered by the porcelain-like incrustation, which I have described in a former lecture; the synovial membrane is not diseased, nor has suppuration ever existed, I believe, within or outside the joint, but the capsular ligament is generally thickened. Many of the symptoms of this disease are common to it, and to morbus coxarius, and hence the necessity of discriminating between them.

The patient first complains of pain about the joint, increased whenever he throws his weight on the affected limb; the limb itself is apparently shortened to the extent of two or three inches; and this, of course, produces considerable lameness, the limb and foot being rotated outwards. The trochanter major is more prominent than in the natural state, the muscles of the thigh are wasted, but not so those of the calf of the leg, according to Mr. Adams at least. The nates of the affected side are quite flat, and no trace exists of the lower fold of the glutæus. Here we have several of the symptoms which attend morbus coxarius; but the points of difference are sufficiently numerous to render our diagnosis easy. In the first place, if you make accurate inquiries touching the history of the case, you will generally discover that the patient experiences some rheumatic pains in other parts of the body, or has previously labored under an attack of rheumatism—in a word, that he is of the rheumatic diathesis. In the next place you will learn, that the pain and stiffness of the joint are relieved by gentle exercise; when the patient has walked about for a short time, the movements of the joint become somewhat more free—a circumstance quite the reverse of what takes place in hip disease, accompanied by shortening, when indeed the patient is totally incapable of walking at all. The pain is not increased by pressing the head of the bone against the acetabulum, and this is a distinctive sign of much value. Finally, on careful examination, you will find that a great deal of the shortening of the limb is apparent, not real; the affected limb may appear to be three inches shorter than the sound one, yet on making accurate measurements of the limb in the manner I have already described, you will discover that two-thirds of the shortening, at least, depend on altered positions of the limb and pelvis. In addition to all this, the constitutional disturbance of true hip disease is never present, nor has the rheumatic affection any tendency to excite the suppurative inflammation which so frequently accompanies the later stages of morbus coxarius. I must, however, observe, that the shortening of the limb in rheumatic cases, which I have just described to you, does not always depend on shortening of the neck of the thigh bone, and deepening of the acetabulum; it may be produced by actual dislocation on the dorsum ilii, and thus is added another remarkable point of resemblance between rheumatic affections of the joint and the ordinary disease.

I have still to consider the treatment of chronic hip disease, but a great deal appertaining to this part of the subject has been already noticed; I shall therefore conclude with a few additional remarks on certain points of treatment which were not sufficiently developed in my preceding lectures. In them I confined my account of treatment to scrofulous affections, what I shall now add is intended to apply to cases of hip disease occurring in persons who are not scrofulous. When speaking of scrofulous affections of joints, I expressed an opinion unfavorable to the use of active antiphlogistic measures in such cases, but this does not apply to hip disease in strong subjects, who present no signs of a scrofulous habit. Here, blood-letting must be employed, or at least blood must be taken by leeches or cupping-glasses from the neighborhood of the joint. For the quantity of blood to be extracted, no fixed rule can be given; you must have regard to the strength and constitutional powers of the patient, his age, the amount of pain, &c., and you must regulate your conduct accordingly. At an early stage of the disease, and in the cases now spoken of, the best general treatment consists in administering calomel, combined with opium, until the mouth is affected. The disease is often arrested as soon as the peculiar effects of mercury manifest themselves; but some patients, as is well known, are unable to bear this powerful remedy, which irritates their bowels, and if continued, may excite dysenteric inflammation of the intestinal mucous membrane. Hence, if pain with tenesmus and scanty mucous stools be observed, an attempt should be made to allay the irritation by opiate injections, and, if these fail, the use of mercury must be immediately abandoned. Counter-irritation may be employed more freely, and at an earlier period in these cases than in scrofulous affections of the hip-joint. When the inflammation has been somewhat subdued by local blood-letting, I generally employ, with advantage, a succession of blisters over the region of the hip. In the second stage, also, counter-irritation must be continued. Some surgeons prefer blisters, while others have recourse to caustic issues behind the trochanter major. When the disease is evidently confined to the osseous tissues, much benefit has been derived from cauterization by means of moxas or the actual cautery. Should any severe inflammatory symptoms appear, we must have recourse to antiphlogistic remedies, and administer purgatives, combined with saline and antimonial medicines. In the cases now alluded to, absolute rest may be enjoined without any great danger of injury to the general health; and hence the patient should be confined to his couch, while the whole limb is kept immovable by a proper apparatus. With respect to other points of treatment, I do not know that anything of importance remains to be noticed. During the third stage of the disease, the same method is applicable to all classes of patients.—*London Lancet.*

